# G6PD testing in support of treatment and elimination of malaria: recommendations for evaluation of G6PD tests

**DOI:** 10.1186/1475-2875-12-391

**Published:** 2013-11-04

**Authors:** Gonzalo J Domingo, Ari Winasti Satyagraha, Anup Anvikar, Kevin Baird, Germana Bancone, Pooja Bansil, Nick Carter, Qin Cheng, Janice Culpepper, Chi Eziefula, Mark Fukuda, Justin Green, Jimee Hwang, Marcus Lacerda, Sarah McGray, Didier Menard, Francois Nosten, Issarang Nuchprayoon, Nwe Nwe Oo, Pongwit Bualombai, Wadchara Pumpradit, Kun Qian, Judith Recht, Arantxa Roca, Wichai Satimai, Siv Sovannaroth, Lasse Vestergaard, Lorenz Von Seidlein

**Affiliations:** 1 PATH, 2201 Westlake Avenue, Suite 200, Seattle, WA 98121, USA; 2 Eijkman Institute for Molecular Biology, Jakarta, Indonesia; 3 National Institute of Malaria Research, New Delhi, India; 4 Eijkman- Oxford Clinical Research Unit, Jakarta, Indonesia; 5 Shoklo Malaria Research Unit, Mae Sot, Thailand; 6 GlaxoSmithKline, Stockley Park, Uxbridge, UK; 7 Australian Army Malaria Institute, Enoggera, Australia; 8 Bill & Melinda Gates Foundation, Seattle, USA; 9 London School of Hygiene and Tropical Medicine, London, UK; 10 President's Malaria Initiative, Greater Mekong Subregion, Bangkok, Thailand; 11 Centers for Disease Control and Prevention; Global Health Group, University of California, San Francisco, USA; 12 University of the State of Amazonas, Manaus, Brazil; 13 Institut Pasteur du Cambodge, Phnom Penh, Cambodia; 14 Mahidol Oxford Research Unit, Bangkok, Thailand; 15 Chulalongkorn University, Bangkok, Thailand; 16 Department of Medical Research, Lower Myanmar, Yangon, Myanmar; 17 Bureau of Vector Borne Diseases, Bangkok, Thailand; 18 PATH, Bangkok, Thailand; 19 Malaria Consortium, Phnom Penh, Cambodia; 20 Thailand Ministry of Public Health, Bangkok, Thailand; 21 National Malaria Control Programme, Phnom Penh, Cambodia; 22 WHO/WPRO, Manila, Philippines; 23 Menzies School of Health Research, Darwin, Australia

## Abstract

Malaria elimination will be possible only with serious attempts to address asymptomatic infection and chronic infection by both Plasmodium falciparum and Plasmodium vivax. Currently available drugs that can completely clear a human of P. vivax (known as “radical cure”), and that can reduce transmission of malaria parasites, are those in the 8-aminoquinoline drug family, such as primaquine. Unfortunately, people with glucose-6-phosphate dehydrogenase (G6PD) deficiency risk having severe adverse reactions if exposed to these drugs at certain doses. G6PD deficiency is the most common human enzyme defect, affecting approximately 400 million people worldwide.

Scaling up radical cure regimens will require testing for G6PD deficiency, at two levels: 1) the individual level to ensure safe case management, and 2) the population level to understand the risk in the local population to guide Plasmodium vivax treatment policy. Several technical and operational knowledge gaps must be addressed to expand access to G6PD deficiency testing and to ensure that a patient’s G6PD status is known before deciding to administer an 8-aminoquinoline-based drug.

In this report from a stakeholder meeting held in Thailand on October 4 and 5, 2012, G6PD testing in support of radical cure is discussed in detail. The focus is on challenges to the development and evaluation of G6PD diagnostic tests, and on challenges related to the operational aspects of implementing G6PD testing in support of radical cure. The report also describes recommendations for evaluation of diagnostic tests for G6PD deficiency in support of radical cure.

## Goals of the G6PD workshop

In October 2012, a workshop in Bangkok, Thailand, brought together researchers, diagnostic test developers, drug developers, National Malaria Control Programme (NMCP) representatives, development partners and donors to discuss priority issues related to malaria treatment [1]. The workshop built upon two previous meetings: a March 2012 meeting in London on the rationale for short-course primaquine in Africa to interrupt malaria transmission [2] and a May 2012 workshop on glucose-6-phosphate dehydrogenase (G6PD) deficiency that was held in South Korea as part of the Asia Pacific Malaria Elimination Network Vivax Working Group annual meeting [3,4]. The Bangkok workshop provided a forum for discussing the knowledge gaps, barriers, and research questions that must be addressed to support broader availability, adoption, and access to G6PD testing in support of radical cure of Plasmodium vivax.

The goals of the Bangkok workshop were to:

1. Identify technical research priorities to support development of appropriate G6PD testing technologies and strategies in support of P. vivax radical cure.

2. Define use case scenarios or malaria treatment-seeking behaviours that a G6PD test or test result must support.

3. Identify operational research priorities to support implementation of appropriate G6PD testing technologies and strategies.

Primaquine can be used at low doses as a malaria gametocytocidal to block the transmission of the parasite to the mosquito, or it can be used at higher doses in longer regimens for radical cure of P. vivax infection. The workshop focused on the use of G6PD testing in support of radical cure. The agenda and selected presentations are available online [1].

### Background and context

G6PD deficiency is the most common human enzyme defect, affecting more than 400 million people worldwide [5]. Several recent reviews have explored the relationship between malaria and G6PD deficiency [4,6-8]. The meeting focused on topics relevant to developing and evaluating in vitro diagnostic tests for G6PD activity.

### Glucose-6-phosphate dehydrogenase

G6PD is a critical housekeeping enzyme in red blood cells that supports protective systems against oxidative challenge by producing the reduced form of nicotinamide adenine dinucleotide phosphate (NADPH). The gene for the G6PD enzyme is spread over 18.5 Kb and 13 exons on the X chromosome and encodes for a 59 KDa polypeptide. The enzyme is active as a dimer or dimer of dimers configuration. G6PD deficiency is manifested in people with reduced levels of intra-erythrocyte G6PD activity arising typically from mutations in the G6PD gene that impact the stability of the enzyme.

Results from several studies suggest that G6PD deficiency may confer some protection not only against severe malaria but also against non-severe disease [9-11]. Indeed, G6PD deficiency prevalence overlaps significantly with current and historical malaria endemicity [12]. Within these populations, the protection conferred by G6PD deficiency may result in a reduced prevalence of G6PD deficiency among malarial patients as compared to the general population [9-11].

### Definition of G6PD activity

One International Unit (U) is the amount of G6PD activity that will convert 1 micromole of NADP + per minute under predetermined substrate and reaction conditions [13]. Activity may be expressed in either a standard number of cells (U/10^12^ red blood cells) or amount of haemoglobin (U/g Hb). G6PD activity is typically determined by measuring G6PD activity in lysate from a whole blood specimen or a red blood cell preparation from a specimen. G6PD deficiency is defined as a less-than-normal level of G6PD enzyme activity in a blood specimen.

Almost 400 allelic variants in the G6PD gene have been recorded [8,14,15]. The variants known to result in G6PD deficiency tend to affect the stability of the enzyme rather than the catalytic activity of the enzyme [7,8,14,15]. G6PD variants are categorized based on the severity of the G6PD deficiency they cause. Class 1 variants cause congenital non-spherocytic haemolytic anaemia. Class 2 variants cause severe enzyme deficiency (less than 10% of normal). Class 3 variants cause moderate to mild enzyme deficiency (10% to 60% of normal). Class 4 variants cause very mild or no enzyme deficiency (60% to 100% of normal) [13,16]. How these activity ranges relate to safety of exposure to 8-aminoquinolines is not very clear, nor is the definition of normal, as discussed below.

### 8-aminoquinolines, malaria, and G6PD deficiency

Primaquine, an 8-aminoquinoline-based drug, is the only available drug recommended by the World Health Organization (WHO) for radical cure of P. vivax infection. The next most advanced product for radical cure is tafenoquine, which recently completed phase 2 clinical trials.

As a radical cure, primaquine is currently used either in a 7 or 14 day regimen in a doses ranging from 0.25-0.5 mg/kg. For patients with mild to moderate variants of G6PD deficiency, a once-per-week, single 0.75 mg/kg dose of primaquine over eight weeks is recommended, although careful monitoring for hemolysis is also recommended. Unfortunately, none of these regimens is operationally easy to implement. In Brazil and Peru, this has been partially addressed by using a higher-dose, shorter-length primaquine regimen. Tafenoquine as a single-dose radical cure therapy would represent a significant advance in P. vivax therapy. However, a major barrier to widescale adoption of both of these drugs is toxicity in people with G6PD deficiency. While all people exposed to primaquine experience some drop in haemoglobin concentrations [17], people with G6PD deficiency are more likely to experience severe haemolysis, leading to severe haemolytic anaemia and, potentially, death. Despite the availability of primaquine since the 1950s, safety data are scarce.

WHO, confronted with emerging resistance to artemisinin and renewed political will to eliminate malaria in many regions of the world, recently released recommendations to administer low doses of primaquine to all patients presenting with falciparum malaria in those settings [18,19]. Based on available data, the new recommended doses are suggested to be low enough to be safe even for G6PD-deficient patients but high enough to have a gametocytocidal effect and block transmission [19,20]. However, before these recommendations can be implemented, primaquine will need to be registered in many countries for this use. Uganda and other countries are conducting studies to better understand local prevalence and types of G6PD deficiency, even within the context of these low doses [2,21].

### User requirements and target product profile for G6PD tests

The possible role of G6PD tests within the context of using primaquine for blocking transmission has been discussed elsewhere [2,18,19]. The Bangkok workshop focused on diagnostic tests for G6PD deficiency in P. vivax case management. One breakout session was dedicated to identifying how a patient typically presents with P. vivax infection, how the patient is managed in this scenario, and what type of diagnostic test would be required to support case management. Scenarios were created for Cambodia, India, Myanmar, and Thailand. At least one national malaria control programme representative participated, along with researchers with experience in each country. The different country groups were asked to select a target patient profile, regardless of whether this type of patient carried the highest burden of disease.

In all four settings, it was determined that the target patient would benefit most from a point-of-care G6PD test. There was robust debate over who would use the test and exactly how far into the periphery of the health system the test should go, depending on how complex the treatment algorithm would be. For many cases, based on the fact that many users would have access to a mobile phone and, therefore, some access to electric power, participants felt that some type of automated reader, while not ideal, may be acceptable. While a reader may restrict some access, it can also confer benefits, such as remote monitoring, and it could possibly support some means of recordkeeping [22]. Part of the Bangkok discussion revolved around how often a G6PD test would have to be performed for each individual, and a discussion arose regarding the challenges of record keeping, especially with migrant populations.

Based on this discussion, workshop participants created a generic target product profile (Table 1) [4].

**Table 1:** Product features of a point-of-care G6PD test in support of radical cure

**Features**	**Ideal**	**Acceptable**	**Comments**
Test output	Binary, deficient/normal	Quantitative	Presumes a consensus definition of normal that aligns with drug safety
User	Village health workers, mobile malaria workers	District hospital, laboratory worker	This will be defined by national malaria control programmes
Platform	Point-of-care similar to a malaria rapid diagnostic test	A disposable device coupled to a portable, battery-operated device; sensitivity significantly better than human eye	A reader would be acceptable if it significantly improves operational performance
Specimen type	Capillary blood	Capillary blood	Tests must be evaluated for performance with this specimen type
Stability requirements	2 years at 37°C	1 year at 37°C	Expect low throughput at clinic level, so requires small quantities per package or long shelf life
Packaging	Maximum 25 tests per kit	Maximum 25 tests per kit	
Operational temperature range	25-40°C	25-40°C	G6PD enzyme activity is highly temperature dependent (see Figure 2)
Operational humidity range	40-90%	40-90%	None.
Time to result	<10 minutes	<30minutes	Availability of the test result should be aligned with malaria diagnosis and treatment work flow
Read window	>1 hour	10 minutes	Ideally, the test result can be read at any time point after the initial time to result
Sensitivity	Detects all patients (100%) with G6PD activity less than a predetermined cut-off, at or less than which it is unsafe to prescribe a particular dosage of an 8-aminoquinoline	>95% for patients at or less than a defined cut-off G6PD activity	For primaquine, where the fluorescent spot test has been accepted as the standard of care, a 30-40% normal G6PD activity cut-off should be used; for new drugs such as tafenoquine, the cut-off is likely to be higher
Specificity	>95%	>70%	It is preferable to have some patients with normal G6PD activity levels classified as deficient as determined by the Receiver Operating Curve of a diagnostic test
Price	Similar to or less than a malaria rapid diagnostic test	Similar to or less than a malaria rapid diagnostic test	G6PD test represents an additional cost over that of malaria diagnosis and treatment

## G6PD product landscape

### G6PD activity tests

A survey of products and reagents available for G6PD deficiency testing shows a surprisingly large number of products in the market (more than 20). Available tests determine the G6PD phenotype and overall G6PD activity in a blood specimen, either by direct measurement or through dyes. The outputs can be quantitative, semi-quantitative, or qualitative depending on the platform and assay. Different types of G6PD phenotype assays have recently been reviewed [4,6,23,24].

When workshop attendees were asked which G6PD tests they use, more than 15 products were mentioned, spanning at least three assay platforms. Perhaps the most consolidated G6PD products are those used for newborn screening, which often have high-complexity and sometimes high-throughput platforms [25]. These tests are used in Southeast Asia in national newborn screening due to the high G6PD deficiency prevalence in the region and the risk for infants to develop severe hyperbilirubinaemia, acute bilirubin encephalopathy, and kernicterus [26,27].

Quantitative tests for G6PD activity are considered the gold standard. Yet the predominant standard of care for G6PD deficiency screening is a qualitative test, the fluorescent spot test, for which there are several commercial kits as well as homebrew assays (assays assembled in the testing laboratory). Beyond those, the wide range of products in the market offer different levels of complexity, usability, and performance. Some of these tests have been developed on platforms more suitable for use within the context of malaria case management [28-33]. Overall, with few exceptions [34], there is a paucity of published data that compare G6PD deficiency determination across platforms, and most products on the market have not been evaluated independently.

### G6PD genotype tests

G6PD genotype tests characterize the genetic contribution to the G6PD phenotype in a patient. There are several levels at which these tests can be performed, with different degrees of accuracy or resolution. Gel electrophoresis or cytochemical staining can indirectly determine zygosity in females based on whether two G6PD proteins with distinct electrophoretic characteristics or two red cell populations with distinct G6PD activity profiles are observed respectively [35-37]. These are predominantly laboratory-based or homebrew assays. More typically, genotyping is performed through polymerase chain reaction (PCR)-based single nucleotide polymorphism (SNP) analysis, and some commercial primer sets are available to determine the genotype through multiplexed PCR. Because not all SNPs can be multiplexed into a single PCR reaction, different panels have been developed based on population prevalence. This genotyping approach is limited to identifying known genotypes and results in severely biased genotype data. Consequently, when both genotyping and phenotyping have been performed on the same patients, the correlation has been mixed [9,38,39]. This is possibly due to different populations experiencing different degrees of polymorphism in this gene and to the severity in G6PD deficiency conferred by the prevalent genotype in a given population.

Sequencing provides the most deterministic G6PD gene characterization, but the G6PD gene—with its 12 introns and 13 exons spanning 18.5 Kb base pairs—is an awkward gene to sequence economically. Given the new sequencing technologies now available, investments should be made in developing multiplexed sequencing assays that look at a range of haemoglobinopathies. Research ethics and consent implications for this type of multiplexed sequencing assay need to be openly investigated and discussed.

### Technical knowledge gaps

To develop G6PD tests that will inform patient management with 8-aminoquinolines, many questions remain to be answered, both in terms of the G6PD assay itself and the clinical context. Most of these questions revolve around two fundamental issues: (1) defining normal G6PD activity, and (2) defining a G6PD activity cut-off greater than which it is safe to administer a drug at a given.

### Defining normal G6PD activity

For the purpose of evaluating diagnostic tests for G6PD deficiency a standard approach for defining an absolute value for normal G6PD activity in a population is required. Ambiguity in how this value is calculated presents practical difficulties in evaluating the performance of G6PD tests, and particularly that of qualitative tests. For qualitative tests, performance will depend on the boundary, or the cut-off point, between normal and deficiency. Typically, G6PD deficiency has been defined as a percentage of normal G6PD activity. In practice, there are almost as many definitions of normal activity as there are publications for evaluating G6PD diagnostic tests [30-33,40,41].

### Defining the boundary between normal G6PD activity and G6PD deficiency

Further complicating the issue, there is a paucity of data to correlate definitions for different degrees of G6PD deficiency with risk after exposure to an 8-aminoquinoline challenge [6,42]. This remains a major knowledge gap in understanding G6PD deficiency and the risk of exposure to primaquine and tafenoquine. While it is known that G6PD genotypes differentially impact the response to primaquine, this knowledge is restricted to only a few of the known G6PD deficiency traits [43,44]. Additionally, acceptable G6PD activity levels for primaquine administration have been defined by the most predominantly used G6PD assay—the fluorescent spot test. This test, by nature of its assay conditions, defines “deficient” at approximately 10% to 30% of normal G6PD activity. As a result, people with severe G6PD deficiency are predominantly excluded from primaquine treatment, whereas most people with mild G6PD activity and most heterozygous women are treated with primaquine. Anecdotally, “this works,” but there are no supportive, published data.

If the goal is to expose only patients with normal G6PD activity to 8-aminoquinolines, then the cut-off G6PD activity level would have to be in range of 60% to 70% of normal values, as per the WHO classification. This would also exclude a significant portion of heterozygous women, at least those in whom there are a significant proportion of G6PD-deficient red blood cells.

**Figure 1:**
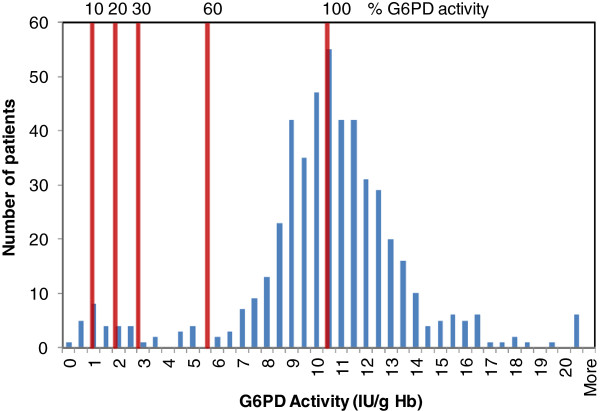
**Histogram of G6PD activity for a population described in Table**3**; 10%, 20%, 30%, and 60% of the adjusted normal G6PD activity for this population are indicated on the graph.**

These two arbitrary definitions or cut-offs have an immense impact on performance requirements for a G6PD test. This is a consequence of the distribution of G6PD activities across a population (Figure 1). Typically, G6PD activity in a population is bimodal, with a minor group of individuals clustered around 10% or less G6PD activity and most clustered in the 60% to 150% range. The 10% to 30% G6PD activity cut-off considered acceptable for primaquine is essentially defined by the fluorescent spot test, a qualitative test for G6PD activity. Thus, developing additional qualitative G6PD tests with similar performance is presumably feasible, though there is a need for improved understanding of the impact of different genotypes on the performance of such qualitative tests against a quantitative test.

By contrast, developing a qualitative G6PD test that accurately excludes patients with less than 60% or 70% G6PD activity is likely to be extremely challenging, given the noise-to-signal levels that are likely to exist at this level of activity. A test with discriminatory capabilities in the 60% to 70% cut-off range is likely to require an underlying quantitative or semi-quantitative platform.

Unfortunately, published G6PD test evaluations use inconsistent definitions of normal G6PD activity and also define test sensitivity and specificity based on different cut-off points or degrees of G6PD deficiency. Thus, it is challenging to understand what a qualitative G6PD test defines as normal or deficient and to compare performance claims between publications. Consistent standards for evaluating G6PD tests are sorely needed.

### Factors affecting G6PD test performance

Several factors can influence the performance of a G6PD test and its ability to correctly classify a patient as either normal or deficient, starting with the cut-off definition as previously described. These include biological conditions such as concomitant haemoglobinopathies, recent haemolytic events that leave a patient with a relatively high proportion of young cells with high G6PD activity that can produce a false normal result, and high leukocyte counts that also lead to a false normal G6PD result. For some of these factors—including a recent malaria infection or other pathological events—it may be possible to predict their effects on a G6PD activity-based assay, but it is still difficult to know how they may affect the risk of an adverse reaction to 8-aminoquinoline exposure. Understanding the impact of haemoglobinopathies and recent haemolytic events on a patient’s response to 8-aminoquinolines and the test performance are critical research questions [4].

**Figure 2:**
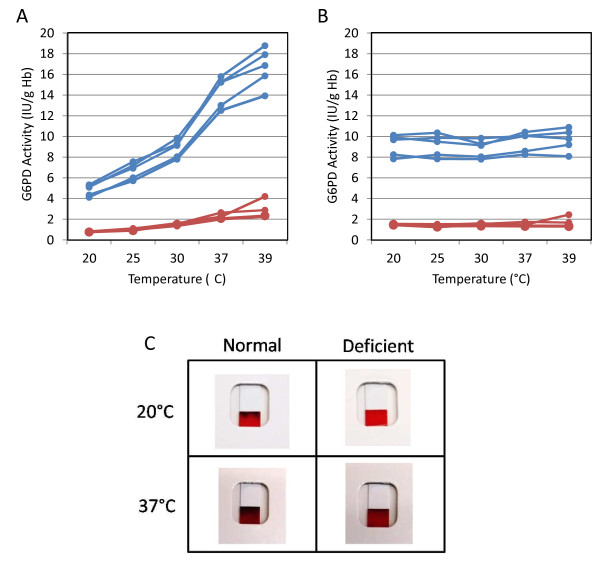
**Impact of temperature on G6PD activity-based tests. A**. Impact of temperature on quantitative determinations of G6PD activity for five normal and four deficient G6PD samples. **B**. Normalization of G6PD activity to 30°C through application of the temperature correction factor (Table 2) to values in **A**. **C**. Impact of temperature on outputs from a qualitative G6PD test. The deficient sample test result at high temperature looks similar to that of a normal sample at low temperature. Note: the temperature range used for Figure 2C is outside the recommended temperature range in the product insert.

Because they are enzyme activity tests, the G6PD assays are particularly sensitive to specimen handling and reaction conditions. Specimen integrity is highly sensitive to handling and storage conditions. Acceptable specimen storage conditions for whole blood is up to 14 days at 4°C and for dried blood spots up to 10 days at 4°C or 48–72 hours at room temperature [28,31,45]. Substrate concentrations and fluctuations in assay temperature influence the enzyme turnover rate. A change of approximately 1 degree in temperature produces a change of 6% in enzyme activity (Figure 2A) [13]. The effect of temperature on G6PD activity values can be accounted for quite effectively by temperature correction factors (Figure 2B). However, in the case of qualitative tests, this may lead to misclassifying deficient specimens as normal if a test is used outside the validated working temperature range (Figure 2C). The combined impact of compromises in specimen collection and operational reaction conditions on the performance of the test in typical malaria treatment settings may result in a wider gap between operational performance of a G6PD test and analytical performance of the test determined under controlled laboratory conditions.

The high proportion of mutations leading to G6PD deficiency affect the stability of the enzyme and specifically the dimer interface [15,46]. Consequently, the dilution factor to which the specimen is subjected in the final assay is also likely to affect the test result and this effect is potentially variant specific (Table 2). Given that the fluorescent spot test is the current standard of care, it will be important to compare the performance of the fluorescent spot test against a quantitative test in different geographical settings to understand this relationship.

**Table 2:** Factor by which blood is diluted in the final G6PD activity assay as performed on different G6PD deficiency diagnostic platforms

	**Trinity biotech G-6-PDH quantitative test**	**R&D diagnostics Ltd quantitative test**	**Trinity biotech G-6-PDH fluorescent spot test**	**Alere BinaxNOW® Malaria test**	**Access Bio CareStart™ G6PD deficiency screening test**
Initial specimen volume	10 ul	5 μl	10 μl	10 μl	3 μl
Dilution factor	301	80	21	8	41

*G*-6-PDH: glucose-6-phosphate dehydrogenase.

In the case of females with heterozygous G6PD alleles, while many display a phenotype of intermediate or mild G6PD deficiency, it is clear from available data that heterozygous women cannot be accurately identified through G6PD enzyme activity assays.

### Proposed principles for evaluating diagnostic tests

G6PD tests play a critical safety role in strategies involving radical cure of P. vivax malaria and there is demand for evaluation of the tests. Defining pragmatic guidelines for the evaluation of G6PD tests will be critical to allow comparison of findings between evaluation studies. Below, one approach which would allow meta-analysis of data across sites is suggested. A quantitative test for the gold standard is recommended, but it is also recognized that it is not trivial to implement a G6PD quantitative assay in many field sites.

### Study population description

Minimal study population characteristics that need to be assessed for any field evaluation include the proportion of G6PD-deficient cases in the study population, mean and median G6PD activity of the study population, and the adjusted male median activity (see below and Table 3). Mean and median values of G6PD activity need to be stratified by gender and adjusted for ambient temperature and the proportion of G6PD-deficient study participants (see below).

**Table 3:** Proposed reference values to describe the G6PD activity profile for a study population

**Reference values**	**Total**	**Female**	**Male**	**Adjusted male**
Number of cases	500	282	218	203
Mean (95% CI) U/g Hb	10.23	10.38	10.03	10.72
Standard deviation	2.28	2.10	2.52	1.97
Median (95% CI) U/g Hb	10.33	10.31	10.34	10.70
Range	0-32.25	0.38-32.25	0-24.32	1.50-24.32

*CI*: confidence interval; *Hb*: haemoglobin; *U*: International Unit.

The table is populated with an example data set randomly selected from a true set of quantitative G6PD test results for a population (data kindly provided by Ari Satyagraha).

If purposive patient recruitment results in inclusion of more G6PD-deficient patients than the local prevalence, mean and median G6PD activity levels also should be provided for the normal males in the study.

### Definitions

The definitions provided below are for performance comparison of a qualitative G6PD test to a quantitative G6PD test.

### Male median

To minimize the impact of heterozygosity on the definition of G6PD activity, researchers should use the median value of G6PD activity for the entire male population in the study. If purposive or biased recruitment were used for an evaluation, the median G6PD value of the G6PD-normal male recruited for the study should be used as the definition of normal. Otherwise, an adjusted male median calculated as described below should be used.

### Adjusted median (100% G6PD activity)

To account for variability in prevalence of G6PD deficiency in a given study population, an adjusted median value is calculated for which males with severe G6PD deficiency (activity less than 10% normal) have been excluded. This is accomplished by:

1. Exclusion of all males with G6PD activity equal to or less than 10% of the male median.

2. Determination of a new median G6PD activity. This is the “adjusted median,” which can be used as the 100% G6PD activity value from which cut-off levels are defined.

### Cut-off

The percentage of adjusted median at or less than which a patient is classified as positive (G6PD deficient). Samples with G6PD activity greater than the cut-off are considered negative.

### True positive (TP)

A sample correctly classified by the diagnostic test under evaluation as having G6PD activity at or less than the cut-off.

### False positive (FP)

A sample incorrectly classified by the diagnostic test under evaluation as having G6PD activity at or less than the cut-off.

### True negative (TN)

A sample correctly classified by the diagnostic test under evaluation as having G6PD activity greater than the cut-off.

### False negative (FN)

A sample incorrectly classified by the diagnostic test under evaluation as having G6PD activity greater than the cut-off.

### Range of patients that should be excluded from treatment with 8-aminoquinolines

All patients with G6PD activity less than or equal to the cut-off as determined by the gold standard test (TP + FN).

### Range of patients with levels of G6PD activity safe to receive treatment with 8-aminoquinolines

All patients with G6PD activity greater than the cut-off (TN + FP).

### Sensitivity

Probability that the test will detect a person with G6PD deficiency.

$$\mathrm{Sensitivity}=\frac{\mathrm{TP}}{\mathrm{TP}+\mathrm{FN}}$$

### Specificity

Probability that the test will detect a person with G6PD-normal activity.

$$\mathrm{Specificity}=\frac{\mathrm{TN}}{\mathrm{TN}+\mathrm{FP}}$$

### Positive predictive value

Probability that the patient is G6PD deficient when the diagnostic test under evaluation yields a positive result.

$$\mathrm{Positive}\;\mathrm{predictive}\;\mathrm{value}=\frac{\mathrm{TP}}{\mathrm{TP}+\mathrm{FP}}$$

### Negative predictive value

Probability that the patient has normal G6PD activity when the diagnostic test yields a negative result.

$$\mathrm{Negative}\;\mathrm{predictive}\;\mathrm{value}=\frac{\mathrm{TN}}{\mathrm{TN}+\mathrm{FN}}$$

### Gold standard testing

An established quantitative G6PD test should be implemented as the gold standard test for which 100% G6PD activity and the cut-offs are defined. The quality of the quantitative test should be controlled either through commercially available artificial controls or through samples with known G6PD activity levels. Ideally, this is performed under strict temperature control using venous blood (acid-citrate-dextrose or EDTA anticoagulant). If strict temperature control cannot be applied, the temperatures at which the assays were performed should be recorded and then standardized to G6PD activity at 30°C according to temperature correction factors. Some product inserts, such as those for the Trinity Biotech. quantitative test, provide temperature correction factors (Table 4).

**Table 4:** Temperature correction factor as provided in the Trinity quantitative spectrophotometric assay product insert

**Cuvette temperature (°C)**	**Temperature correction factor**	**Cuvette temperature (°C)**	**Temperature correction factor**
20	1.90	30	1.00
21	1.76	31	0.94
22	1.66	32	0.89
23	1.55	33	0.83
24	1.46	34	0.78
25	1.37	35	0.74
26	1.28	36	0.70
27	1.20	37	0.66
28	1.13	38	0.62
29	1.06	39	0.58

### Sample size calculations for diagnostic test evaluation

The sample size for evaluations of G6PD tests is driven by the expected performance of the diagnostic test against the predicate gold standard, the local G6PD deficiency prevalence, and the desired accuracy for resulting sensitivity and specificity claims (width of 95% confidence intervals around estimates of sensitivity and specificity). Given the relatively low G6PD deficiency prevalence in most populations worldwide, the sample size is primarily driven by the prevalence and desired accuracy for the evaluation results. Table 5 shows sample calculations for a set of expected test sensitivities over two accuracy constraints and for three G6PD deficiency prevalence rates. In the absence of an appropriate sample size, the statistical power of the study is compromised and the implied uncertainty of the study must be clearly explained.

**Table 5:** Sample size calculations for evaluation of G6PD diagnostic tests for radical cure

**Expected sensitivity**	**Desired width of CI**	**Confidence level**	**Number of disease cases needed**	**Sample size**		
				**Prevalence rate 10%**	**Prevalence rate 15%**	**Prevalence rate 20%**
0.8	0.06	0.95	715	7150	4767	3575
0.8	0.1	0.95	264	2640	1760	1320
0.9	0.06	0.95	417	4170	2780	2085
0.9	0.1	0.95	158	1580	1053	790
0.95	0.06	0.95	238	2380	1587	1190
0.95	0.1	0.95	94	940	627	470
0.96	0.06	0.95	200	2000	1333	1000
0.96	0.1	0.95	81	810	540	405
0.97	0.06	0.95	161	1610	1073	805
0.97	0.1	0.95	68	680	453	340
0.98	0.06	0.95	123	1230	820	615
0.98	0.1	0.95	55	550	367	275
0.99	0.06	0.95	87	870	580	435
0.99	0.1	0.95	44	440	293	220

*CI*: confidence interval.

### G6PD test performance criteria

In the absence of a more complete understanding of the relationship between risk of haemolysis and level of G6PD deficiency, as well as local G6PD reference values, it is impossible to define a clear normal/deficient G6PD activity cut-off that is consistent and clinically relevant as pertaining to safety and treatment with an 8-aminoquinoline. As a consequence, test performance criteria should be provided for a range of G6PD activity. Percentage of median activity is proposed in order to account for inter-assay and inter-laboratory variability in absolute G6PD activity values. The minimum proposed degrees of deficiency are based on WHO classifications and commonly used ranges: 10%, 20%, 30%, and 60% of the normal male or adjusted median G6PD activity. Absolute cut-off values (in U/g Hb) and sensitivity, specificity, positive predictive value, and negative predictive value should be determined for this range of degrees of G6PD deficiency. Example performance data for the evaluation of a putative G6PD test are described in Tables 3 and 6; the cut-offs are shown in Figure 1.

**Table 6:** **Performance results for a putative qualitative diagnostic test modeled against the quantitative results described in Table**3

	**10% cut-off**	**20% cut-off**	**30% cut-off**	**60% cut-off**
Cutoff value (U/g Hb)	1.07	2.14	3.21	6.42
Number of samples with G6PD levels less than cut-off (percentage)	14 (2.8)	24 (4.8)	28 (5.6)	41 (8.2)
Sensitivity percentage (95% CI)	100	95.8	89.3	68.3
	(73–100)	(77–100)	(71–97)	(52–81)
Specificity percentage (95% CI)	97.1	98.9	99.4	100
	(95–98)	(97–100)	(98–100)	(99–100)
Positive predictive value percentage (95% CI)	0.5	0.82	0.89	1.00
	(0.31-0.69)	(0.62-0.93)	(0.71-0.97)	(0.84-1.00)
Negative predictive value percentage (95% CI)	1.00	1.00	0.99	0.97
	(0.99-1.00)	(0.99-1.00)	(0.98-1.00)	(0.95-0.98)

*CI*: confidence interval; *Hb*: haemoglobin; *U*: International Unit.

### Regulatory considerations for G6PD testing

The first step toward regulating the quality of G6PD tests will be to define evaluation standards for this class of diagnostic tests. In many countries where G6PD tests are needed to support P. vivax case management, regulatory mechanisms for diagnostic tests are absent, weak, or in transition. In the absence of national guidelines, some countries default to CE mark and US Food and Drug Administration (FDA) approval. Currently, the BinaxNOW® G6PD test marketed in the United States has obtained FDA approval under 510(k) clearance. Most G6PD tests on the market have at best obtained only CE mark approval.

There is a concern that without clear guidelines for G6PD testing performance criteria, point-of-care G6PD testing will follow a similar route as the malaria rapid diagnostic tests (RDTs), albeit to a smaller scale, wherein a large number of products with varying degrees of quality control and performance entered the market. Variability in RDT quality produced distrust of the product generally, and slowed uptake of RDT technology. For G6PD tests, prevention, rather than remediation, of such a problem will likely be less costly for the malaria control and elimination community.

### Operational considerations for G6PD testing

Although participants in the workshop’s use case scenario session unanimously identified a point-of-care G6PD test as the ideal product profile to support P. vivax case management with 8-aminoquinolines, it does not necessarily follow that:

1. This product profile has a large market demand. The workshop attendees were primarily focused on malaria patients who are the hardest to reach rather than on the largest number of people at risk.

2. This is the best solution for all use cases. As neonatal screening programmes improve in many countries, a more cost-effective approach may be to improve information management systems such that the G6PD status of a patient is more readily available and the need for repeat testing can be minimized.

In Malaysia, neonatal G6PD screening is routinely performed, and G6PD records accompany the patient. In a case where a patient’s status is not known, a fluorescent spot test is done, and primaquine is administered based on G6PD status. In contrast, in the Philippines, neonatal screening is supposed to be routinely done but is not universally available, especially to remote and indigenous populations most at risk of malaria infection.

The goal of operational research around G6PD deficiency testing and radical cure with 8-aminoquinolines should focus on how to ensure that G6PD status information is available at the point of case management for a patient presenting with P. vivax infection. This may involve linking drug availability to availability of a point-of-care G6PD test, to medical records, or a combination of the two.

Another challenge with introducing and scaling up new G6PD tests is that there are currently few guidelines for adopting and training end users on G6PD testing and counseling. Also, confirming or evaluating operational effectiveness of a G6PD test in clinical settings, as opposed to analytical performance, will be challenging. Additionally an external quality assurance programme will be required. Cost analysis of different approaches to ensuring safe delivery of 8-aminoquinolines should take these factors into consideration, as they may significantly influence cost-effectiveness outcomes.

Market studies segmenting where point-of-care G6PD tests are needed in place of more complex assays will be useful for malaria programmes in terms of resource allocation and for suppliers in terms of understanding the true market size. From the pricing perspective, ideally a G6PD test would be available at the price of a malaria RDT or less. For primaquine, given its low cost, a significantly more expensive test will shift the burden of the cost significantly from treatment costs to diagnostic costs and may impact willingness to pay. Potentially more expensive drugs may tolerate higher prices. From a programme perspective, cost-effectiveness studies should be designed to identify boundaries of these costs.

## Conclusions

From a public health perspective, uncertainty remains on whether G6PD testing deficiency status does not need to be taken into account for primaquine-based radical cure in some populations, as reflected in the current WHO guidelines. However, from a patient management perspective, where the individual risk/benefit ratio dictates optimal treatment, knowing the G6PD status of the patient is a prerequisite for prescribing an 8-aminoquinoline-based drug.

Although many questions remain regarding G6PD deficiency and the risk of drug-related adverse events, this should not hinder efforts to evaluate and adopt G6PD tests in support of radical cure. G6PD testing represents an additional cost for malaria treatment and unnecessary G6PD testing should be minimized. Health systems, health management information systems, care-seeking practices, and malaria epidemiology will determine the best way to ensure knowledge of G6PD status for people who have access to 8-aminoquinoline radical cure regimens. While an approach that includes population screening and effective recordkeeping is attractive for the long term, it is clear that point-of-care G6PD testing will be required to meet immediate needs, given that the populations most at risk of P. vivax infection are typically those at the periphery of health care systems and the hardest to reach. In these scenarios, significant operational research will be required to understand how to supply these tests, who the end users should be, how to link the availability of the tests with that of the drugs, and how to implement a recordkeeping system that minimizes the need for repeat testing of individual patients.

A prerequisite to introducing G6PD testing is the availability of high-quality G6PD tests with product profiles that are compatible with end-use cases. Establishing pragmatic and consistent criteria for evaluation of tests should be a high priority. The development and evaluation of new G6PD tests can benefit from the availability of specimen panels [47]. Because factors unique to local populations may affect the performance of G6PD tests, another priority should be to understand the impact of geographical and genetic diversity on the performance of these tests.

## Abbreviations

CI: Confidence interval; FDA: US Food and drug administration; FN: False negative; FP: False positive; G6PD/G-6-PDH: Glucose-6-phosphate dehydrogenase; Hb: Haemoglobin; PCR: Polymerase chain reaction; RDT: Rapid diagnostic test; SNP: Single nucleotide polymorphism; TN: True negative; TP: True positive; U: International unit; WHO: World health organization.

## Competing interests

The authors declare that they have no competing interests.

## Authors’ contributions

GJD wrote the first draft of the manuscript. All authors contributed to the content, read and approved the final manuscript. LSV is a staff member of the World Health Organization. The author alone is responsible for the views expressed in this publication and they do not necessarily represent the decisions or policies of the World Health Organization.
